# Agriculture–urban interfaces, social vulnerability and climate change shape West Nile virus risk across the United States

**DOI:** 10.1098/rsbl.2025.0817

**Published:** 2026-10-07

**Authors:** Samantha B. Sambado, Váleri N. Vasquez, Mauricio Cruz-Loya, Johannah E. Farner, Rachel L. Fay, Samantha J. Bents, Joshua E. Lazaro, Talya Shragai, Isabel Delwel, Desire U. Nalukwago, Erin Mordecai

**Affiliations:** ^1^ Department of Biology, Stanford University, Stanford, CA, USA; ^2^ Center for International Security and Cooperation, Stanford University, Stanford, CA, USA; ^3^ Department of Medicine, Division of Infectious Diseases and Geographic Medicine, Stanford University, Stanford, CA, USA; ^4^ Center for Human and Planetary Health, Stanford Woods Institute for the Environment, Stanford University, Stanford, CA, USA; ^5^ Microbiology and Immunology Department, Stanford University, Stanford, CA, USA; ^6^ Stanford University, Stanford, CA, USA

**Keywords:** *Culex* mosquitoes, vector-borne disease, temperature-driven transmission, climate warming, land use heterogeneity, zoonotic disease risk, West Nile virus

## Abstract

Climate and land use change are reshaping the dynamics of vector-borne diseases. West Nile virus (WNV), the most widespread zoonotic arbovirus in the United States, illustrates the need to integrate climate, land cover and social vulnerability across heterogeneous landscapes when assessing spatial risk. We present a nationwide, county-level assessment of WNV risk using complementary statistical and mechanistic models to (i) identify socioecological correlates of current WNV incidence and (ii) project vector species-specific, temperature-dependent transmission suitability under mid- and late-century climate change scenarios. We found that land cover gradients, temperature-driven transmission and both occupational and residential exposure are associated with WNV incidence, particularly in mixed urban–agricultural landscapes. Future temperature and land cover projections suggest spatially variable shifts in environmental risk driven by divergent physiological responses among *Culex* species vectors. Our results highlight temperature and land cover as consistent, mechanistically grounded correlates of WNV risk at the national scale, while underscoring the need for refined, mosquito species-specific analyses at local levels. These insights can inform more targeted surveillance, vector control and climate adaptation strategies. We also identified key knowledge gaps, particularly around host and vector ecology, which must be addressed to improve public health responses in the face of ongoing environmental change.

## 1. Introduction

As a widespread climate-sensitive virus transmitted across diverse ecosystems and species, West Nile virus (WNV) serves as a model system for understanding how global changes reshape zoonotic vector-borne disease risk [1,2]. Its transmission involves multiple mosquito vectors and diverse avian hosts, occurring across heterogeneous environments ranging from urban centres to agricultural areas [3–5] ( figure 1). Consequently, WNV risk to humans varies spatially and temporally, even within endemic regions [7–10]. Assessing spatial risk requires considering the multi-faceted nature of transmission, including environmental suitability for the enzootic cycle and factors influencing spillover to humans, especially since reported cases often underestimate true infection rates owing to mild infections [7,11]. Integrating these components enables more effective targeting of surveillance and adaptation efforts, particularly in regions experiencing rapid environmental change [12].

**Figure 1.**
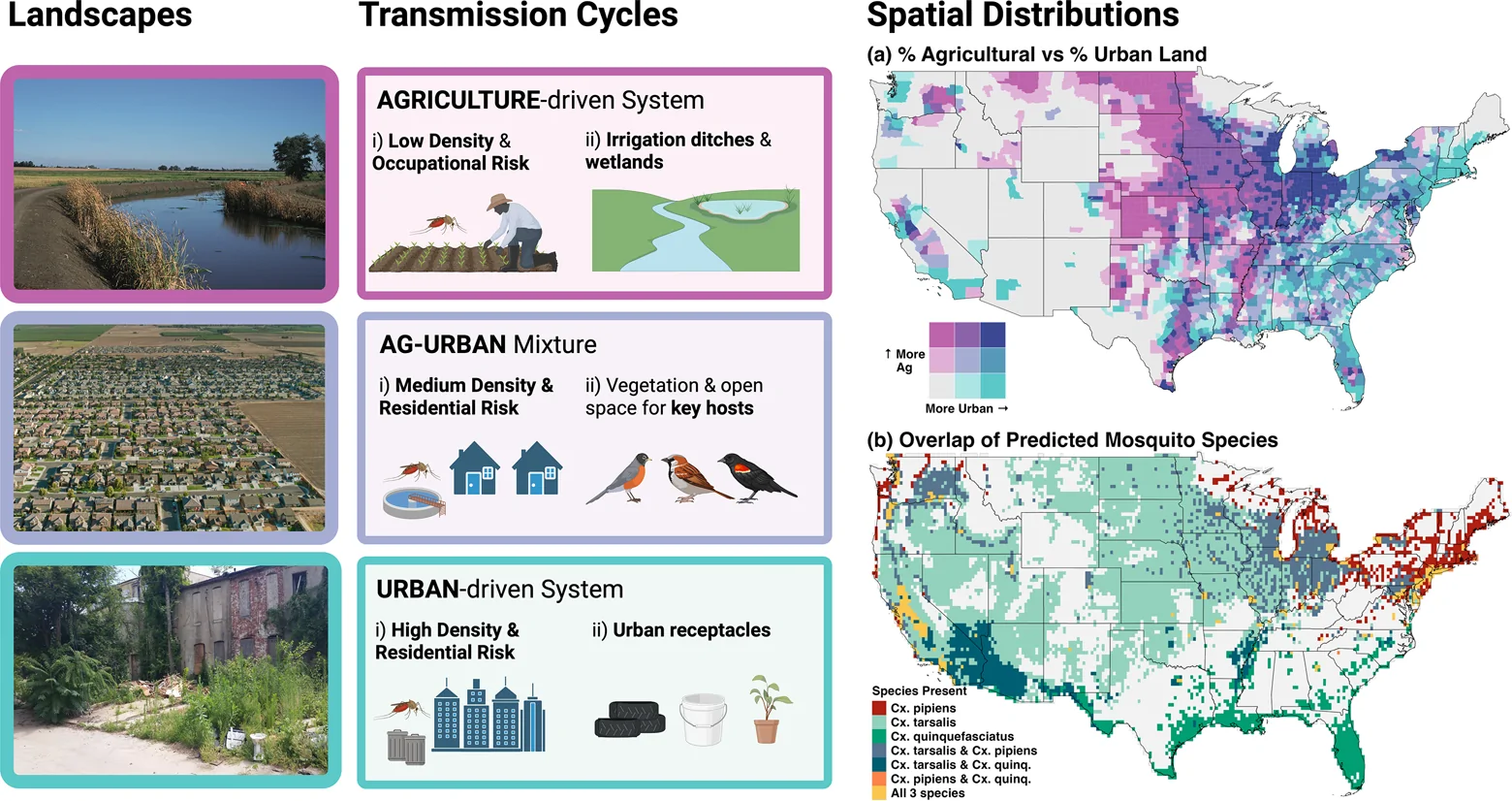
Conceptual model of WNV transmission dynamics. Transmission risk is shaped by (i) human density and (ii) mosquito–virus amplification dynamics, influenced by (a) land cover and (b) *Culex* species distributions. In (b), a probability threshold of ≥0.6 was used to classify pixels as occupied by a given mosquito species [6]. Created with BioRender, Sambado, S (https://BioRender.com/z5ck3wg). Photographs (top, centre and bottom) are credited to Gary Kramer (courtesy of USDA Natural Resources Conservation Science), Hal Bergman (courtesy of Getty Images) and the Baltimore Ecosystem Study LTER Photo Archive, respectively.

Previous studies have highlighted the importance of mosquito ecology [13,14], environmental drivers [2,15] and social determinants [16,17] of WNV risk, but these factors are often examined either within localized systems or through models that prioritize predictive performance using a large number of potentially correlated covariates. Additionally, humans are often treated as endpoints rather than components of ecological systems, despite their role as dead-end spillover hosts, whose exposure to mosquitoes varies across occupational and residential contexts. Land cover, for example, is hypothesized to influence vector–host contact rates for both humans and birds, but its effects are mediated through context-dependent ecological processes. In agricultural areas, standing water from irrigation can create ideal breeding sites for *Culex tarsalis*, potentially increasing occupational exposure for outdoor workers [7,9,18]. These same irrigation ditches and waterways can also attract passerine birds, which serve as amplifying hosts for WNV [19,20]. By contrast, urban environments may pose greater residential exposure to *Culex pipiens*, particularly where lower-quality housing facilitates mosquito breeding or indoor entry [21,22]. Urbanization is also broadly associated with reduced bird diversity, an ecological shift that may increase human WNV risk if the birds that persist in low-diversity settings are also competent hosts [23,24]. However, these coarse diversity–disease patterns may not capture the complex, localized interactions between vector species and bird species of varying competence and attractiveness to vectors [14,25,26]. As a result, while land cover and related variables are biologically motivated and widely used, their ability to explain spatial variation in WNV incidence at a continental scale remains uncertain—particularly across heterogeneous ecological and social contexts, such as transitional zones where urban and agricultural land covers intersect and ecological interactions may become more complex [27,28].

Temperature is another key and quantifiable driver of WNV transmission, influencing mosquito and viral traits that collectively determine transmission potential [29–31]. Mechanistic models of temperature-dependent transmission (i.e. *R_0_*(*T*)) capture these nonlinear relationships and can be paired with high-resolution climate and land cover projections to anticipate future environmental hotspots of disease transmission [2,30–32]. In the USA, where multiple *Culex* species co-occur but have distinct temperature responses for transmission, species-specific modelling can clarify how warming may differentially affect vector populations [6,30]. Because climate change is unlikely to affect all species uniformly, incorporating species-specific biology into risk assessment is helpful for capturing the complexity of future disease dynamics.

In this study, we addressed the challenge of identifying generalizable drivers of WNV risk at a continental scale by adopting a parsimonious, hypothesis-driven modelling framework. Specifically, we (i) integrated diverse socioecological variables to identify nonlinear correlates of current WNV incidence and (ii) projected *Culex* species-specific, temperature-driven transmission suitability under mid- and late-century climate and land cover change scenarios. Our county-level results offer spatially explicit insights into how landscape structure and temperature interact to shape WNV dynamics across the contiguous USA. In doing so, we highlight the strategic priorities for empirical research to advance our understanding of how ecological processes can guide proactive, geographically targeted public health surveillance.

## 2. Methods

We summarize the primary modelling approaches to quantify landscape features, host–vector contact rates and climate variability that influence WNV transmission. Full methods are provided in S1 and S2, and tables S1 and S2 in the supplementary material.

### (a) Human West Nile virus data

County-level incidence data were obtained from ArboNET, the national surveillance system. For counties with reported cases, we used the average annual incidence of WNV neuroinvasive disease (WNND) per 100 000 residents from 1999 to 2023. Counties with no case data were excluded from the analysis. Neuroinvasive cases were used owing to their higher likelihood of diagnosis and reporting, as these cases involve more severe clinical presentations, including meningitis [33].

### (b) Covariates

#### (i) Vector habitat

We obtained county-level land cover data from the 2020 National Land Cover Database (NLCD). NLCD codes 81 and 82 were categorized as ‘agricultural land’ and codes 21–24 as ‘urban land’. To quantify the spatial interaction between urban and agricultural areas, we calculated the urban–agricultural edge density (m km^−^^2^) for each county [34]. Higher edge density values represent a more intermixed urban–agricultural landscape, whereas lower values represent a more homogeneous land cover. For future mosquito habitat suitability, we used projected land cover from the USGS FORE-SCE model [35] for agricultural and developed land types and assigned species-specific suitability values for *Cx. pipiens*, *Cx. tarsalis* and *Culex quinquefasciatus* based on known habitat associations [6].

#### (ii) Human exposure

Understanding human exposure to mosquitoes requires accounting for the diverse environmental contexts in which people inhabit, including occupational and residential settings. For occupational exposure, we focused on agricultural workers, who are likely at an elevated risk owing to prolonged outdoor activity in landscapes favourable to *Cx. tarsalis* [17]. We estimated occupational exposure using county-level employment data from the Bureau of Labor Statistics Quarterly Census of Employment and Wages (QCEW). We calculated the proportion of agricultural workers relative to total private-sector employment per county. Although these data likely underestimate employment, the QCEW remains the best available agricultural employment source [36]. To capture potential mosquito exposure within the home environment, complementing outdoor occupational risk, we incorporated proxies that may facilitate mosquito entry, particularly for *Cx. pipiens*. Specifically, we used the CDC's Social Vulnerability Index (SVI), which captures demographic and socioeconomic characteristics at the county level [37]. Higher SVI values reflect greater vulnerability, including poverty and related social determinants that may elevate mosquito exposure risk (supplementary material, S3).

#### (iii) Bird hosts

We used the geographic ranges of 20 bird species with the highest WNV competence (based on infection studies [38]) from the USGS Gap Analysis Program to estimate the number of competent bird species present at the county level.

#### (iv) Climate

To estimate future climate conditions, we used temperature projections from the CMIP6 GFDL-CM4 global climate model, which was selected for its suitability to our study region [39]. These projections were statistically downscaled to approximately 6 km resolution using LOCAv2 [40]. To capture multiple possible futures for the USA, we analysed two Shared Socioeconomic Pathways (SSPs): SSP2-4.5, a moderate-emission, middle-of-the-road pathway; and SSP5-8.5, a high-emission, fossil-fuelled development pathway. While RCP8.5 has historically been interpreted as a ‘business-as-usual’ scenario, recent research suggests that it represents an unlikely emission trajectory [41,42]. We included SSP5-8.5 to explore the upper-bound climate impacts. Climate projections were assessed for three time periods: the current period (2015–2020), mid-century (2045–2074) and late-century (2075–2100).

#### (v) Temperature-dependent transmission

We estimated the temperature-dependent transmission suitability using a mechanistic, trait-based reproductive number (*R_0_*(*T*)) model for *Cx. pipiens*, *Cx. tarsalis* and *Cx. quinquefasciatus* based on Mordecai *et al.* [43] and parametrized with WNV-specific traits from Shocket *et al.* [30] (supplementary material, S2, table S2). The model incorporates temperature-dependent mosquito and viral traits, producing relative *R_0_*(*T*) values scaled 0–1 to indicate thermal suitability (hereafter referred to as *R_0_*(*T*)). To calculate annual county-level *R_0_*(*T*) values, we first calculated the peak-season *R_0_*(*T*) values for each of the three *Culex* species within a focal county. To do so, for each species, we averaged the monthly *R_0_*(*T*) values from the peak *Culex* activity window (April through October), accounting for potential temperature-independent diapause that occurs outside of this seasonal period. These peak-season *R_0_*(*T*) values for the three *Culex* species in a given county were then averaged to obtain a final county-level *R_0_*(*T*) value. For temperature inputs into the *R_0_*(*T*) model, we used downscaled CMIP6 temperature projections for the current, mid- and late-century periods under these two emission scenarios. We then quantified changes in thermal suitability by calculating the mean difference in *R_0_*(*T*) between the current and future time periods across emission scenarios for each vector species.

### (c) Analysis

#### (i) Current correlates of West Nile virus

To examine how land cover, bird host dynamics and human exposure contribute to WNV incidence, we fitted a generalized additive model (GAM) using the county-level average annual incidence of WNND as the outcome (supplementary material, S2). Significant county-level covariates included urban–agricultural edge density, total competent bird species present and proxies for human exposure (i.e. occupational and residential risk). Relative *R_0_*(*T*), based on county-level temperature data and averaged across *Culex* species, was also included as a covariate. To account for demographic differences in the risk of severe disease, we included the percentage of the population aged ≥ 65 years. All covariates were standardized prior to fitting. The outcome, WNND incidence, was log_10_-transformed. To account for spatial autocorrelation, we included Gaussian process smooths for the latitude and longitude of county centroids. Individual covariate significance was assessed using the ‘gam.hp’ package [44]. See S2 in the supplementary material for model justifications and diagnostics.

#### (ii) Projected correlates of West Nile virus

To estimate the potential for environmental WNV transmission under future climate scenarios, we developed a composite risk metric by integrating temperature-dependent transmission potential with projected mosquito habitat suitability, representing environmentally constrained transmission suitability (hereafter referred to as environmental risk). Specifically, we multiplied the species-specific *R_0_*(*T*) estimates, which were each scaled to a range from 0 to 1, with habitat suitability layers derived from the USGS FORE-SCE land use projections for developed and agricultural areas [35]. Environmental risk values were assigned based on established habitat associations for each *Culex* species, as detailed in S2.3 in the supplementary material. This approach produced a scaled, spatially explicit risk metric for each species, where higher values represented areas with greater environmental risk for WNV transmission under mid- and late-century climate and land cover conditions. This approach identifies areas where both competent vector presence and thermally permissive transmission conditions overlapped. Our analysis is limited to agricultural and urban land types, as these are the most ecologically relevant to the focal *Culex* species and are among the most reliably projected components of future land use change in the USA [45]. We acknowledge that this simplification was a key assumption and limitation. As such, the resulting maps are intended to support visualization and exploratory spatial assessment rather than directly predict human disease incidence or replace localized epidemiological modelling efforts.

All analyses were conducted using RStudio version 4.4.1 [46]. Data cleaning and visualizations were conducted using ‘tidyverse’ and ‘ggplot2’ packages [47,48]. Original data sources are publicly available and are detailed in table S3 in the supplementary material.

## 3. Results

### (a) Land cover, climate and social vulnerability associate with current human West Nile virus incidence

We found that county-level incidence of WNND was significantly associated with both environmental and human exposure factors (figure 2, table 1). While demographic (age ≥ 65) and spatial (latitude and longitude) controls were significant covariates, our hypothesized covariates also showed consistent and ecologically plausible associations with WNV incidence. Based on the percentage of deviance explained in the full model (%), individual covariates were ranked in relative importance as follows: county longitude and latitude (75.6%), average peak season *R_0_*(*T*) (7.69%), urban–agricultural edge density (5.37%), agricultural employment (3.13%), social vulnerability index (SVI, 3.02%), percentage of population aged ≥65 (2.97%) and total competent bird species (2.21%). The final model, based on 2105 county observations, explained 72.3% of the total deviance. The large effects of spatial smoothing terms are expected, given their phenomenological flexibility to capture unobserved local variations in WNV risk, which may be driven by local variations in the transmission cycle and human risk. Partial effect plots revealed nonlinear relationships among the smooth terms, highlighting the threshold values and offering directions for future research. The clearest threshold emerged for *R_0_*(*T*) (effective degrees of freedom, e.d.f. = 7.89) and SVI (e.d.f. = 2.92), where e.d.f. = 1 indicates a linear relationship, and higher values reflect increasing nonlinearity. Predicted WNV incidence trended positively with *R_0_*(*T*) but declined beyond a threshold at the upper end of the *R_0_*(*T*) range, which may reflect the upper temperature limit for *Culex* species. Risk also increased sharply when SVI exceeded approximately 0.75. As a reference, counties with SVI near this threshold that are also densely populated include Queens in NY, Milwaukee in WI and DeKalb in GA (supplementary material, S2). These findings suggest that socially vulnerable communities may face a disproportionately higher WNV incidence. However, this index encompasses multiple dimensions of vulnerability (e.g. poverty and housing); therefore, the underlying mechanisms driving this threshold remain unclear (supplementary material, S3). The relationship for urban–agricultural edge density (e.d.f. = 6.08) was also strongly nonlinear but less intuitive. Risk for WNV was highest in low edge densities, indicating that a homogeneous land cover—whether predominantly urban, agricultural or another type—may promote transmission. There was a noticeable additional peak at intermediate values, suggesting an increased risk in transitional zones where urban and agricultural land covers mix. Other variables had clear linear relationships with WNND incidence; agricultural employment was positively associated, while the total number of competent bird species showed a negative relationship, potentially reflecting a disconnect between bird species richness and species evenness, which may contribute more directly to the transmission cycle. These findings underscore the need for mechanistic studies that also account for temporal dynamics such as bird migration. Our results suggested that current WNV transmission risk is influenced by nonlinear and interacting relationships between socioecological factors.

**Figure 2.**
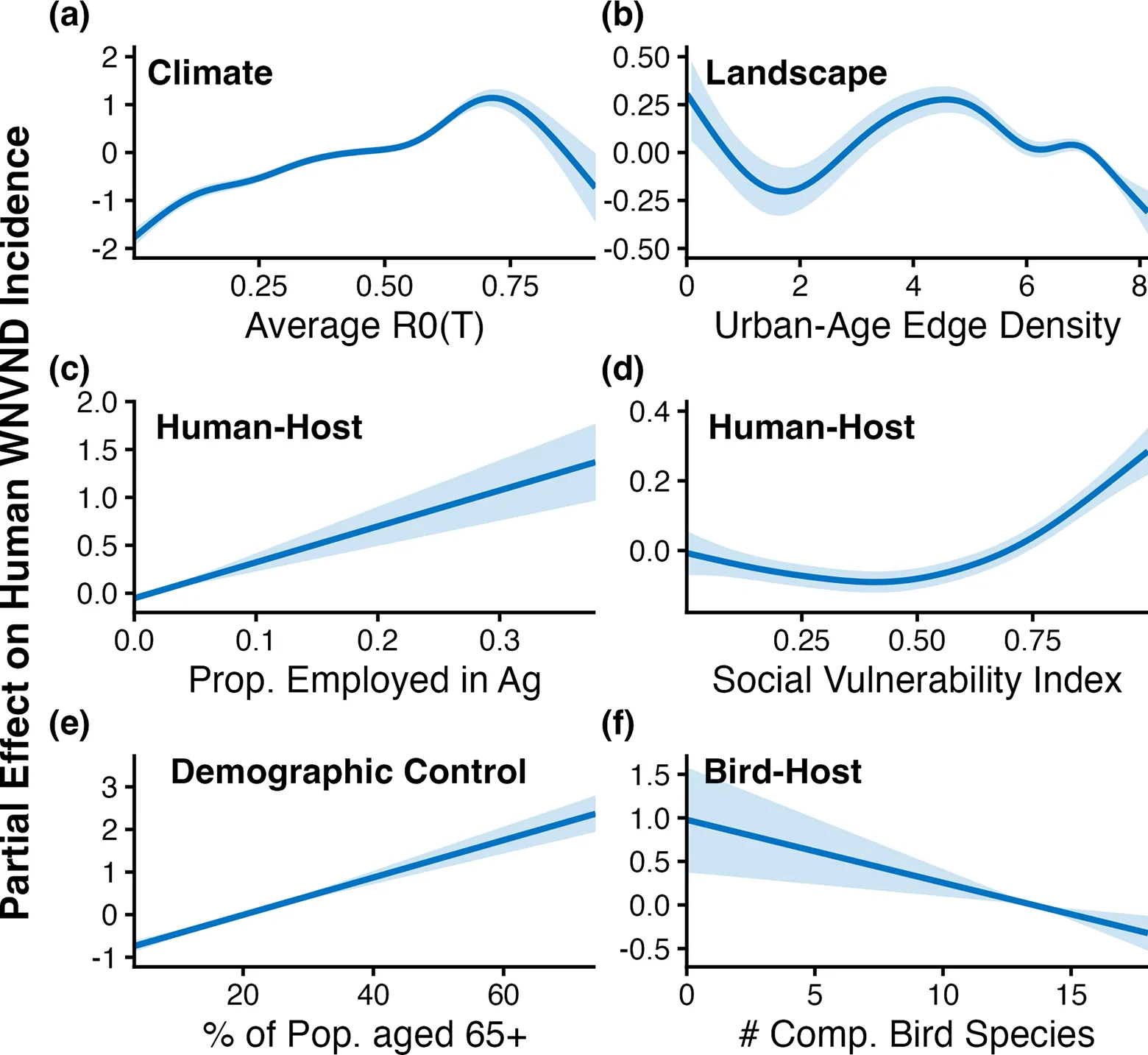
Socioecological correlates of current WNV incidence. Partial effects from a GAM, ordered by importance (a–f), illustrate associations between county-level WNND incidence and covariates (Ag: agriculture). The shaded areas represent 95% confidence intervals.

**Table 1.** GAM results for county-level WNND incidence. The percentage of deviance explained (%) indicates the proportion of the model's deviance attributed to each covariate, as estimated using the ‘gam.hp’ package. Note that all covariates were initially modelled as splines, but any covariates with e.d.f. < 2 (indicating a relationship that is approximately linear) were then fitted as linear terms. Linear terms are reported as estimated coefficients with standard errors (SE), *t*-values and *p*-values. Smooth terms are summarized by estimated degrees of freedom (e.d.f.), reference degrees of freedom (d.f.), *F*-statistic and *p*-values.

covariate	estimate	SE	*t*-value	*p*-value	%
intercept	–0.79	0.023	–34.23	< 2.00 × 10^–16^	
prop. employed in Ag	0.12	0.019	6.68	3.17 × 10^–11^	3.34
% of population aged ≥65	0.22	0.020	10.86	< 2.00 × 10^–16^	2.97
total competent bird species	–0.22	0.068	–3.18	0.002	2.14
	**e.d.f.**	**d.f.**	***F*-statistic**	***p*-value**	**%**
s(longitude, latitude)^b^	27.58	19.17	109.43	< 2.00 × 10^–16^	75.61
s(average peak-season *R_0_*(*T*))^a^	7.89	8.68	20.00	< 2.00 × 10^–16^	7.94
s(urban–agricultural edge density)^a^	6.09	7.27	4.64	3.22 × 10^–5^	5.58
s(social vulnerability index)^a^	2.92	3.65	6.82	5.67 × 10^–5^	3.02

Model fit: *n* = 2105; adjusted *R*^2^ = 0.72; deviance explained = 72.3%.

a Cubic spline smooth.

b Gaussian process smooth.

### (b) Projected temperature-driven and focal land cover changes in West Nile virus transmission

Temperature-driven transmission suitability, measured by *R_0_*(*T*), and urban–agricultural edge density emerged as the strongest mechanistic covariates in the GAM (i.e. excluding the spatial smoothing term) and are both projected to increase under most climate and land use scenarios. However, the magnitude of change varies by mosquito species and geographic region. Across all US counties and *Culex* species, average *R_0_*(*T*) values are projected to either increase or remain stable from current to future periods under both emission scenarios (table 2). *Culex pipiens* showed the largest overall gain in average thermal suitability, with its mean relative *R_0_*(*T*) increasing by 0.21 (on a scale from 0 to 1). By contrast, *Cx. quinquefasciatus* and *Cx. tarsalis* exhibited more modest gains of 0.15 and 0.06, respectively. Notably, under the high-emission scenario, both *Cx. quinquefasciatus* and *Cx. tarsalis* showed declines in mean *R_0_*(*T*) from mid- to late-century, indicating potential thermal constraints at elevated temperatures. By contrast, *Cx. pipiens* continues to gain thermal suitability across both future periods, reaching its peak average *R_0_*(*T*) under late-century high-emission conditions. Spatial projections of environmental risk (figure 3) for urban and agricultural areas revealed more nuanced vector species-specific responses, which we expand upon in the discussion. These responses underscore the importance of integrating both climate and land use projections when anticipating future WNV risk, as environmental suitability for vectors will not shift uniformly across landscapes.

**Table 2.** Average temperature-driven transmission suitability, *R_0_*(*T*) values, across all continental US counties during peak transmission season (April through October) for each *Culex* species under current and future climate scenarios. Projections are for mid-century (2045–2074) and late-century (2075–2100) under two emission scenarios (SSP2-4.5 and SSP5-8.5). Values (mean ± standard deviation) reflect species-specific thermal suitability across all counties, based on CMIP6 climate model estimates.

		SSP2-4.5 (intermediate-emission)		SSP5-8.5 (high-emission)	
**species**	current	mid	late	mid	late
***Cx. pipiens***	0.36 ± 0.22	0.45 ± 0.23	0.49 ± 0.22	0.50 ± 0.22	0.57 ± 0.19
***Cx. tarsalis***	0.48 ± 0.22	0.53 ± 0.20	0.48 ± 0.18	0.54 ± 0.17	0.51 ± 0.15
***Cx. quinquefasciatus***	0.40 ± 0.22	0.46 ± 0.21	0.55 ± 0.18	0.48 ± 0.18	0.47 ± 0.15

**Figure 3.**
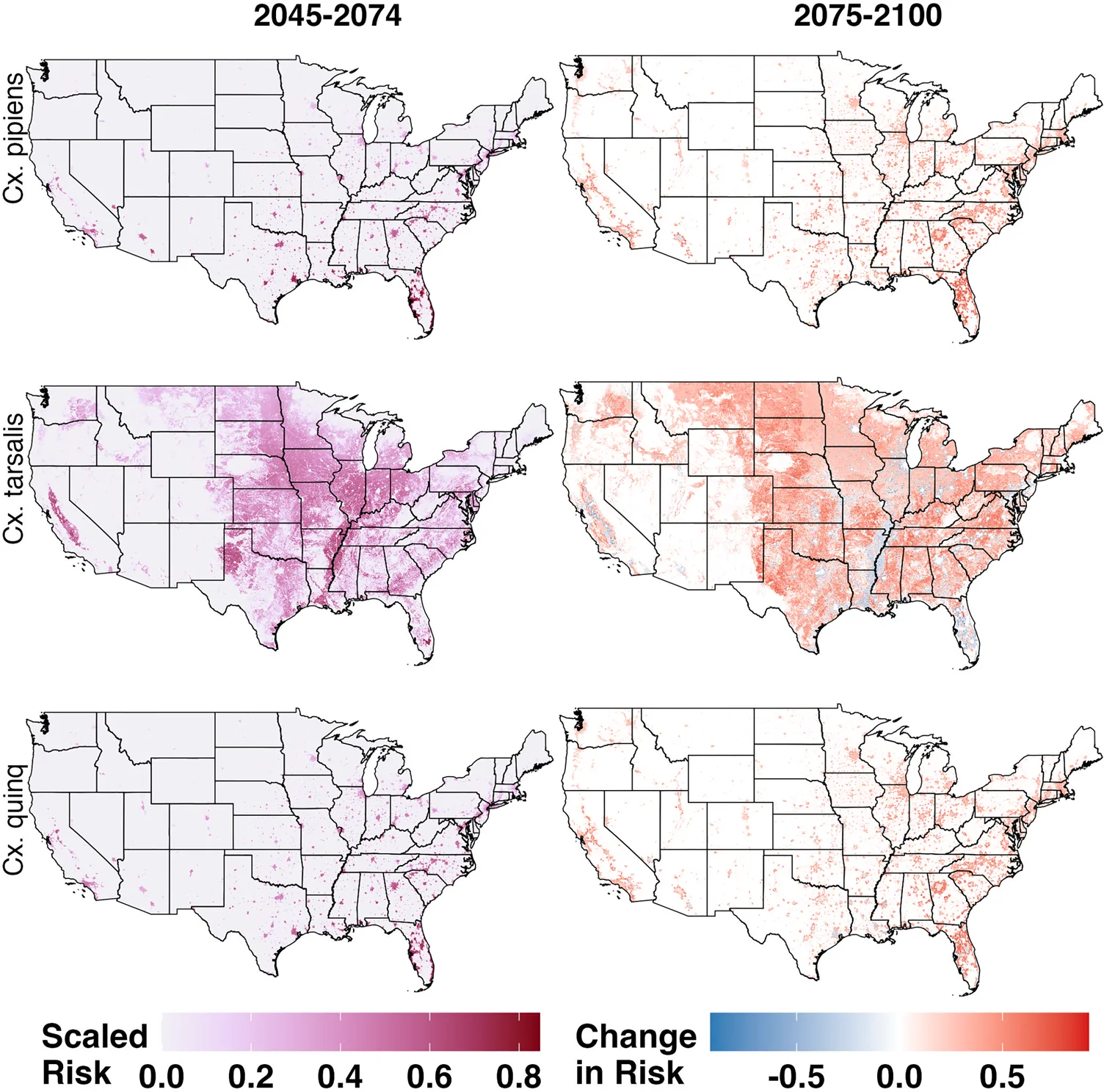
Spatial patterns of projected WNV risk. Projected scaled risk under future, temperature and land-use types, based on forecasted land covers from the USGS FORE-SCE model [35] and future *R_0_*(*T*) estimates under the SSP2-4.5 scenario for mid-century (2045–2074) and late-century (2075–2100). Projections focus only on urban and agricultural land cover classes. Changes in risk are shown relative to 2045–2074 baseline period. Rows represent *Cx. pipiens*, *Cx. tarsalis* and *Cx. quinquefasciatus* in the top, middle and bottom rows, respectively.

## 4. Discussion

As climate and land use change accelerate, understanding the ecological and social factors that shape WNV transmission is increasingly critical for anticipating and mitigating emerging public health threats. Our study demonstrated that temperature and landscape structure, particularly homogeneous or transitional zones between urban and agricultural areas, have a consistent but complex relationship with WNV risk. We suggest that these mixed-use environments support overlapping distributions of vectors, birds and humans, especially in vulnerable communities [4,27,28,49]. By investigating current socioecological indicators alongside projected vector species-specific, land cover and temperature-dependent transmission models, we present a biologically grounded and complementary framework for understanding disease risk in a changing environment.

Our models showed that land use heterogeneity, suitable temperatures for transmission and human exposure collectively contribute to the spatial variation in WNND incidence across US counties. While earlier studies have identified elevated risk in semi-urban or peri-urban zones, our work is among the first to examine national-scale edge effects, supporting prior regional results that WNND incidence is elevated in mixed environments [21,27,28,49]. We hypothesized that these mixed zones combine suitable habitats, moderately competent bird hosts and high enough human population density to drive focal WNV hotspots [25,27]. We also found high risk in more homogeneous landscapes, likely mirroring the importance of occupational and socioeconomic factors [16–18]. Agricultural employment, a proxy for outdoor occupational exposure to *Cx. tarsalis*, showed a positive association with WNND incidence, although further research is needed to understand underreporting and access to care in these populations [17]. In urban settings, our findings align with established associations between socioeconomic disadvantage and WNND incidence [16,17,22], potentially mediated by local conditions, such as poor drainage, waste accumulation, inadequate housing structures and limited vector control, which are all conducive to *Cx. pipiens* proliferation [22,50]. While our social vulnerability index detected a risk threshold, its aggregated nature limits mechanistic interpretation (supplementary material, S4). Sub-county analyses incorporating specific housing metrics (e.g. infrastructure age, air conditioning access) would likely improve explanatory power, especially in counties with high inequality [16,51]. Prior insights from economically diverse Orange County, California, underscore the importance of incorporating fine-scale socioeconomic and infrastructure into WNND incidence models, as disparities in infrastructure maintenance, such as the prevalence of neglected swimming pools, can significantly amplify disease risk in lower-income communities [16]. Our bird-related findings align with previous research, suggesting that a greater richness of competent bird species may be associated with reduced human WNV incidence [23,52]. However, our bird metric is limited in that it does not account for seasonal abundance or host-feeding preferences of *Culex* mosquitoes under natural conditions; for example, while both American robins and American crows are competent hosts in laboratory settings, robins are more frequently fed upon by *Culex* mosquitoes in the field [26]. We highlight the host-feeding preference of *Culex* species as an important area for future research to aid in our understanding of WNV dynamics (table 3).

**Table 3.** Future research priorities to improve spatial models of WNV risk under climate and land use changes.

1.	**Refine the spatial and temporal scale** of the analysis with more finely resolved epidemiological data, to identify predictors of locally elevated risk.
2.	**Refine bird-host metrics** by incorporating *Culex* species’ feeding preferences and temporally resolved bird data (e.g. eBird, Breeding Bird Survey) to better capture avian amplification dynamics within a season and across years.
3.	**Develop mechanistic residential exposure metrics** at a finer spatial scale (e.g. data from American Community Survey, including housing quality, infrastructure age, swimming pool presence and air conditioning access) to better assess mosquito entry into households (i.e. residential risk), particularly in socially vulnerable communities.
4.	**Integrate land cover and population projections** into transmission models to anticipate how urban expansion and demographic changes will reshape future WNV exposure patterns.
5.	**Model water dispersal patterns** on the landscape, including the effects of irrigation and urban stormwater infrastructure, to better identify where mosquito breeding habitats form and persist.
6.	**Establish a standardized, nationwide database of *Culex* abundance** that is readily publicly available using data from counties with active vector control programmes, to support consistent and scalable vector surveillance.
7.	**Account for evolutionary responses** of *Culex* vectors and WNV to climate change, including potential shifts in thermal tolerance and vector competence under warming conditions.
8.	**Incorporate the impacts of variation in WNV exposure and immunity** in humans and bird populations.

Future WNV risk is unlikely to be spatially uniform, as each *Culex* species and important bird hosts respond differently to climate and land use change owing to distinct thermal physiologies and habitat preferences [53]. While the national averages of temperature-dependent transmission potential suggest broad trends over time (table 2), these summaries can obscure important regional heterogeneity. Spatial projections of environmental risk (figure 3) across urban and agricultural areas reveal nuanced, vector species-specific patterns. Although much of the nation's agricultural land is projected to remain thermally suitable, *Cx. tarsalis,* which has the broadest thermal tolerance (20.1°C), may experience localized declines in habitat suitability, particularly in regions such as the Central Plains, where agricultural land is projected to be converted. *Culex quinquefasciatus*, common in the southern USA, has the narrowest thermal breadth (12.7°C) and is expected to see reduced thermal suitability in parts of the south, including current endemic hotspots such as California and Arizona, where future temperatures may exceed its upper thermal limit (31.8°C) [13]. *Culex pipiens*, which has the highest upper thermal limit (34.9°C) and is widely distributed in northern urban areas, is projected to expand its range, particularly in areas undergoing urban development [31,32,54,55]. These mosquito species-specific responses emphasize the importance of incorporating both climate and land use projections when assessing future WNV risk, as environmental risk depends on the interaction between temperature thresholds and habitat availability. While our projections assume fixed thermal limits, evolutionary adaptation or behavioural plasticity in mosquitoes could shift these outcomes over time [56]. The resulting county-level risk maps, which integrate temperature-dependent transmission and vector habitat suitability, provide a spatially explicit tool to support targeted surveillance and vector control under future environmental change.

From a public health perspective, our findings highlight further research directions to identify strong mechanistic links, particularly those involving social vulnerability and occupational exposure, which can inform local interventions. Our results suggest that improving infrastructure in residential areas and addressing occupational risks could meaningfully reduce environmental WNV exposure, even as climate suitability increases in the USA [57]. Practical interventions, such as improving water management, upgrading housing infrastructure and conducting outreach to at-risk occupational groups, especially agricultural workers, may help mitigate spillover in ecologically suitable regions [16,51]. Targeted management of agricultural landscapes also offers opportunities to reduce risk, for example, by optimizing irrigation practices to minimize standing water and reduce mosquito breeding habitats, particularly in arid regions where irrigation can sustain vector populations well beyond the rainy season [8,9,58]. As urbanization continues to reshape the American landscape, there is a real risk that WNV transmission could be exacerbated without intentional, proactive planning [24,45]. Newly urbanized areas, particularly in the Central Great Plains, are of special concern for *Cx. pipiens*-driven transmission, given their overlap with existing WNV hotspots [4,11,18]. As projected declines in agricultural land cover may reduce habitat suitability for *Cx. tarsalis*, concurrent urbanization and warming temperatures are likely to favour *Cx. pipiens*, indicating a shift in dominant transmission pathways rather than a uniform change in overall environmental suitability. In these regions, urban design that limits mosquito breeding habitat can reduce disease risk [27,50,57]. Our results show the value of integrating socioecological and climatic data to develop forward-looking, locally grounded public health strategies in the face of ongoing environmental change.

While our study offers novel insights into the spatial ecology of WNV at the county-level scale across the USA, several caveats and limitations must be acknowledged. First, our analyses parse the risk of WNND incidence at the county level on average across the period since the virus has invaded the USA—a scale too coarse to capture both local relationships among land cover, birds, vectors and humans and weather anomalies that drive temporal variation. This likely explains the dominance of the spatial term and weaker model performance in regions such as the Rocky Mountains and Central Plains, where temporally dynamic processes (e.g. water availability and bird migration) play a key role. Although drought is an important climatic driver of WNV risk, it was not significant in our final analysis. This is likely because we included spatial controls that encompass local drought variability. Drought could play a more important role in models that explicitly incorporate its temporal variability and use finer (sub-county) spatial resolution to represent vector–host dynamics [2,58]. Notably, our static bird metric does not capture seasonal dynamics of bird migration, which is important for focal WNV hotspots [25,26]. Mosquito feeding behaviour is another critical consideration (supplementary material, S1); however, empirical data remain limited and highly localized. In addition, our use of the social vulnerability index, while informative for hypothesis generation, cannot identify mechanisms of exposure. We examined more targeted indicators that directly relate to physical conditions (supplementary material, S3) but found the county scale to be too coarse to capture the localized influence of these variables. Finally, our future projections assume a limited number of climate scenarios and no changes in bird or human exposure metrics, which introduce uncertainty; therefore, our results should be interpretative as relative, not absolute, risk estimates.

Our findings highlight the value of mechanistic, landscape-informed models that integrate species ecology and socioeconomic context, as well as unique regional factors, which are important to understand WNV incidence. Because the majority of WNV infections are subclinical, scalable socioecological frameworks are essential for proactive prediction and intervention. Advances in high-resolution climate and land use projections, when paired with local metrics of human exposure, can refine vector control strategies under future environmental changes. Continued investment in interdisciplinary approaches will be essential to reduce the burden of WNV and other vector-borne diseases.

## Supplementary Material



## Data Availability

Original data sources are publicly available and detailed in table S3. Codes to recreate figures and analysis are stored on the Dryad repository [59]. Supplementary material is available online [60].
