# Supplementary material for: Agriculture–urban interfaces, social vulnerability and climate change shape West Nile virus risk across the United States
Source: Biol Lett. 2026 Oct 7;22(10):20250817. doi: 10.1098/rsbl.2025.0817 (PMC13641919; doi:10.1098/rsbl.2025.0817)
Supplement: Supplementary file 1 [file rsbl.2025.0817.zip › rsbl.2025.0817/rsbl20250817_si_002.docx]

**Title:** Agriculture-urban interfaces, social vulnerability, and climate change shape West Nile virus risk across the United States

**Authors:** Samantha Sambado^1*^, Váleri N. Vasquez^1,2^, Mauricio Cruz-Loya^1^, Johannah E. Farner^1^, Rachel L. Fay^1^, Samantha Bents^1, 3^, Joshua E. Lazaro^1^, Talya Shragai^4^, Isabel O. Delwel^5^, Desire I. Uwera Nalukwago^1^, and Erin A. Mordecai^1^

**Affiliations:**

1. Department of Biology, Stanford University, Stanford, California, United States of America
2. Center for International Security and Cooperation, Stanford University, Stanford, California, United States of America
3. Division of Infectious Diseases and Geographic Medicine, Department of Medicine, Stanford University, Stanford, California, United States of America
4. Center for Human and Planetary Health, Stanford Woods Institute for the Environment, Stanford, California, United States of America
5. Department of Microbiology and Immunology, Stanford University, Stanford, California, United States of America

***Corresponding author:** Samantha Sambado ([ssambado@stanford.edu](mailto:ssambado@stanford.edu))

**Abstract**

Climate and land use change are reshaping the dynamics of vector-borne diseases. West Nile virus (WNV), the most widespread zoonotic arbovirus in the United States, illustrates the need to integrate climate, land cover, and social vulnerability across heterogenous landscapes when assessing spatial risk. We present a nationwide, county-level assessment of WNV risk, using complementary statistical and mechanistic models to (1) identify socio-ecological correlates of current WNV incidence, and (2) project vector species-specific, temperature-dependent transmission suitability under mid- and late-century climate change scenarios. We find that land cover gradients, temperature-driven transmission, and both occupational and residential exposure are associated with WNV incidence, particularly in mixed urban-agricultural landscapes. Future temperature and land cover projections suggest spatially variable shifts in environmental risk, driven by divergent physiological responses among *Culex* species vectors. Our results highlight temperature and land cover as consistent, mechanistically grounded correlates of WNV risk at the national scale, while underscoring the need for refined, mosquito species-specific analyses at local levels. These insights can inform more targeted surveillance, vector control, and climate adaptation strategies. We also identify key knowledge gaps, particularly around host and vector ecology, that must be addressed to improve public health response in the face of ongoing environmental change.

Keywords: *Culex* mosquitoes, vector-borne disease, climate warming, West Nile virus, land cover change

**1. Introduction**

As a widespread, climate-sensitive virus transmitted across diverse ecosystems and species, West Nile virus (WNV) serves as a model system for understanding how global change reshapes zoonotic vector-borne disease risk (1,2). Its transmission involves multiple mosquito vectors and diverse avian hosts, occurring across heterogeneous environments ranging from urban centers to agricultural areas (3–5) (Figure 1). Consequently, WNV risk to humans varies spatially and temporally, even within endemic regions (6–9). Assessing spatial risk requires considering the multifaceted nature of transmission, including environmental suitability for the enzootic cycle and factors influencing spillover to humans – especially since reported cases often underestimate true infection rates due to mild infections (6,10). Integrating these components enables more effective targeting of surveillance and adaptation efforts, particularly in regions experiencing rapid environmental change (11).

Previous studies have highlighted the importance of mosquito ecology (12,13), environmental drivers (2,14), and social determinants (15,16) of WNV risk, but these factors are often examined either within localized systems or through models that prioritize predictive performance using large number of potentially correlated covariates. Additionally, humans are often treated as endpoints rather than components of ecological systems, despite their role as dead-end spillover hosts whose exposure to mosquitoes varies across occupational and residential contexts. Land cover, for example, is hypothesized to influence vector-host contact rates with both humans and birds, but its effects are mediated through context-dependent ecological processes. In agricultural areas, standing water from irrigation can create ideal breeding sites for *Culex tarsalis*, potentially increasing occupational exposure for outdoor workers (6,8,17). These same irrigation ditches and waterways can also attract passerine birds, which serve as amplifying hosts for WNV (18,19). In contrast, urban environments may pose greater residential exposure to *Culex pipiens*, particularly where lower-quality housing facilitates mosquito breeding or indoor entry (20,21). Urbanization is also broadly associated with reduced bird diversity, an ecological shift that may increase human WNV risk if the birds that persist in low-diversity settings are also competent hosts (22,23). However, these coarse diversity–disease patterns may not capture complex, localized interactions between vector species and bird species of varying competence and attractiveness to vectors (13,24,25). As a result, while land cover and related variables are biologically motivated and widely used, their ability to explain spatial variation in WNV incidence at continental scales remains uncertain - particularly across heterogeneous ecological and social contexts, such as transitional zones where urban and agricultural land covers intersect and ecological interactions may become more complex (26,27).

Temperature is another key and quantifiable driver of WNV transmission, influencing mosquito and viral traits that collectively determine transmission potential (28–30). Mechanistic models of temperature-dependent transmission (i.e., R_0_(T)) capture these nonlinear relationships and can be paired with high-resolution climate and land cover projections to anticipate future environmental hotspots of disease transmission (2,29–31). In the United States (US), where multiple *Culex* species co-occur but have distinct temperature responses for transmission, species-specific modeling can clarify how warming may differentially affect vector populations (29,32). Because climate change is unlikely to affect all species uniformly, incorporating species-specific biology into risk assessment is helpful for capturing the complexity of future disease dynamics.

In this study, we address the challenge of identifying generalizable drivers of WNV risk at a continental scale by adopting a parsimonious, hypothesis-driven modeling framework. Specifically, we (1) integrate diverse socio-ecological variables to identify nonlinear correlates of current WNV incidence, and (2) project *Culex* species-specific, temperature-driven transmission suitability under mid- and late-century climate and land cover change scenarios. Our county-level results offer spatially explicit insights into how landscape structure and temperature interact to shape WNV dynamics across the contiguous US. In doing so, we highlight strategic priorities for empirical research to advance our understanding of how ecological processes can guide proactive, geographically targeted public health surveillance.

**2. Methods**

We summarize the primary modeling approaches to quantify landscape features, host-vector contact rates, and climate variability that influences WNV transmission. Full methods are provided in the Supplement (Text S1-2, Tables S1-2).

(a) Human WNV data

County-level incidence data were obtained from ArboNET, the national surveillance system. For counties with reported cases, we used the average annual incidence of WNV neuroinvasive disease (WNND) per 100,000 residents from 1999-2023. Counties with no case data were excluded from the analysis. Neuroinvasive cases were used due to their higher likelihood of diagnosis and reporting because cases involve more severe clinical presentations including meningitis (33).

(b) Covariates

*Vector habitat*: We obtained county-level land cover data from the 2020 National Land Cover Database (NLCD). NLCD codes 81 and 82 were categorized as “agricultural land”, and codes 21-24 as “urban land”. To quantify the spatial interaction between urban and agricultural areas, we calculated the urban-agricultural edge density (m/km^2^) for each county (34). Higher edge density values represent a more intermixed urban-agricultural landscape, while lower values represent more homogenous land cover. For future mosquito habitat suitability, we used projected land cover from the USGS FORE-SCE model (35) for agricultural and developed land types, and assigned species-specific suitability values for *Cx. pipiens*, *Cx. tarsalis*, and *Culex quinquefasciatus* based on known habitat associations (32).

*Human exposure*: Understanding human exposure to mosquitoes requires accounting for the diverse environmental contexts people inhabit, including occupational and residential settings. For occupational exposure, we focus on agricultural workers, who are likely at elevated risk due to prolonged outdoor activity in landscapes favorable to *Cx. tarsalis* (16). We estimated occupational exposure using county-level employment data from the Bureau of Labor Statistics Quarterly Census of Employment and Wages (QCEW). We calculated the proportion of agricultural workers relative to total private-sector employment per county. Although these data likely underestimate employment, QCEW remains the best available agricultural employment source (36). To capture potential mosquito exposure within the home environment, complementing outdoor occupational risk, we incorporated proxies that may facilitate mosquito entry, particularly for *Cx. pipiens*. Specifically, we used the CDC’s Social Vulnerability Index (SVI), which captures demographic and socioeconomic characteristics at the county level (37). Higher SVI values reflect greater vulnerability, including poverty and related social determinants that may elevate mosquito exposure risk (Text S3).

*Bird hosts*: We used the geographic ranges of 20 bird species with the highest WNV competence (based on infection studies (38)) from the USGS Gap Analysis Program to estimate the number of competent bird species present at the county level.

*Climate:* To estimate future climate conditions, we used temperature projections from the CMIP6 GFDL-CM4 global climate model, selected for its suitability to our study region (39). These projections were statistically downscaled to ~6 km resolution using LOCAv2 (40). To capture multiple possible futures for the US, we analyzed two Shared Socioeconomic Pathways (SSPs): SSP2-4.5, a moderate-emission, middle-of-the-road pathway, and SSP5-8.5, a high-emission, fossil-fueled development pathway. While RCP8.5 has historically been interpreted as a “business-as-usual” scenario, recent research suggests it represents an unlikely emission trajectory (41,42). We include SSP5-8.5 to explore the upper-bound climate impacts. Climate projections were assessed for three time periods: the current period (2015-2020), mid-century (2045-2074), and late-century (2075-2100).

*Temperature-dependent transmission*: We estimated temperature-dependent transmission suitability using a mechanistic, trait-based reproductive number (R_0_(T)) model for *Cx. pipiens*, *Cx. tarsalis*, and *Cx. quinquefasciatus*, based on Mordecai et al. (2013) (43) and parameterized with WNV-specific traits from Shocket et al. (2020) (29) (Text S2, Table S2). The model incorporates temperature-dependent mosquito and viral traits, producing relative R_0_(T) values scaled 0-1 to indicate thermal suitability (hereafter referred to as R_0_(T)). To calculate annual county-level R_0_(T) values, we first calculated peak-season R_0_(T) values for each of the three *Culex* species within a focal county. To do so, for each species, we averaged monthly R_0_(T) values from the peak *Culex* activity window (April through October), accounting for potential temperature-independent diapause that occurs outside of this seasonal period. This peak-season R_0_(T) values for the three *Culex* species in a given county were then averaged at a final county-level R_0_(T) value. For temperature inputs into the R_0_(T) model, we use downscaled CMIP6 temperature projections for current, mid-, and late-century periods under two emission scenarios. We then quantified changes in thermal suitability by calculating the mean difference in R_0_(T) between current and future time periods across emission scenarios for each vector species.

(c) Analysis

*Current correlates of WNV*: To examine how land cover, bird host dynamics, and human exposure contribute to WNV incidence, we fit a generalized additive model (GAM) using county-level average annual incidence of WNND as the outcome (Text S2). Significant county-level covariates included urban-agricultural edge density, total competent bird species present, and proxies for human exposure (i.e., occupational and residential risk). Relative R_0_(T), based on county-level temperature data and averaged across *Culex* species, was also included as a covariate. To account for demographic differences in the risk of severe disease, we included the percentage of the population aged ≥65. All covariates were standardized prior to fitting. The outcome, WNND incidence, was log_10_-transformed. To account for spatial autocorrelation, we included Gaussian process smooths for the latitude and longitude of county centroids. Individual covariate significance was assessed using the `gam.hp` package (44). See Text S2 for model justifications and diagnostics.

*Projected correlates of WNV*: To estimate the potential for environmental WNV transmission under future climate scenarios, we developed a composite risk metric by integrating temperature-dependent transmission potential with projected mosquito habitat suitability, representing environmentally constrained transmission suitability (hereafter referred to as environmental risk). Specifically, we multiplied the species-specific R_0_(T) estimates, which are each scaled to range from 0-1, with habitat suitability layers derived from the USGS FORE-SCE land-use projections for developed and agricultural areas (35). Environmental risk values were assigned based on established habitat associations for each *Culex* species, as detailed in Text S2.3. This approach produced a scaled, spatially explicit risk metric for each species, where higher values represent areas with greater environmental risk for WNV transmission under mid- and late-century climate and land cover conditions. This approach identifies areas where both competent vector presence and thermally permissive transmission conditions overlap. Our analysis is limited to agricultural and urban land types, as these are the most ecologically relevant to the focal *Culex* species and are among the most reliably projected components of future land-use change in the US (45). We acknowledge that this simplification is a key assumption and limitation. As such, the resulting maps are intended to support visualization and exploratory spatial assessment, rather than to directly predict human disease incidence or replace localized epidemiological modeling efforts.

All analyses were conducted in RStudio version 4.4.1 (46). Data cleaning and visualizations were conducted with `tidyverse` and `ggplot2` packages (47,48). Original data sources are publicly available and detailed in Table S3. Code to recreate figures and analysis are stored on Dryad repository (doi:10.5061/dryad.dz08kps9j) (49).

**3. Results**

*(a) Land cover, climate, and social vulnerability associate with current human WNV incidence*

We found that county-level incidence of WNND was significantly associated with both environmental and human exposure factors (Figure 2, Table 1). While demographic (age ≥ 65) and spatial (latitude and longitude) controls were significant covariates, our hypothesized covariates also showed consistent and ecologically plausible associations with WNV incidence. Based on the percentage of deviance explained in the full model (%), individual covariates were ranked in relative importance as follows: county longitude and latitude (75.6%), average peak season R_0_(T) (7.69%), urban-agricultural edge density (5.37%), agricultural employment (3.13%), social vulnerability index (SVI, 3.02%), percent of population aged ≥65 (2.97%), and total competent bird species (2.21%). The final model, based on 2,105 county observations, explained 72.3% of the total deviance. The large effects of spatial smoothing terms is expected given their phenomenological flexibility to capture unobserved local variation in WNV risk, which may be driven by local variation in the transmission cycle and human risk. Partial effect plots revealed non-linear relationships among the smooth terms, highlighting threshold values and offering directions for future research. The clearest threshold emerged for R_0_(T) (effective degrees of freedom, edf = 7.89) and SVI (edf = 2.92), where edf =1 indicates a linear relationship, and higher values reflect increasing non-linearity. Predicted WNV incidence trended positively with R_0_(T) but declined beyond a threshold at the upper end of the R_0_(T) range, which may reflect the upper temperature limit for *Culex* species. Risk also increased sharply when SVI exceeded ~0.75. As a reference, counties with SVI near this threshold that are also densely populated include Queens, NY; Milwaukee, WI; and DeKalb, GA (Text S2). These findings suggest that socially vulnerable communities may face disproportionately higher WNV incidence. However, this index encompasses multiple dimensions of vulnerability (e.g., poverty, housing), so the underlying mechanisms driving this threshold remain unclear (Text S3). The relationship for urban-agricultural edge density (edf = 6.08) was also strongly non-linear, but less intuitive. Risk for WNV was highest in low edge densities, indicating that homogenous land cover – whether predominantly urban, agricultural, or another type – may promote transmission. There was a noticeable additional peak at intermediate values, suggesting increased risk in transitional zones, where urban and agricultural land cover mix. Other variables had clear linear relationships with WNV incidence: agricultural employment was positively associated, while the total number of competent bird species showed a negative relationship, potentially reflecting a disconnect between bird species richness and species evenness, which may contribute more directly to the transmission cycle. These underscore the need for mechanistic studies that also account for temporal dynamics. Together, these results suggest that current WNV transmission risk is influenced by nonlinear and interacting relationships between socio-ecological factors.

*(b) Projected temperature-driven and focal land cover changes in WNV transmission*

Temperature-driven transmission suitability, measured by R_0_(T), and urban-agricultural edge density emerged as the strongest mechanistic covariates in the GAM (i.e., excluding the spatial smoothing term) and are both projected to increase under most climate and land-use scenarios. However, the magnitude of change varies by mosquito species and geographic region. Across all US counties and *Culex* species, average R_0_(T) values are projected to either increase or remain stable from current to future periods under both emission scenarios (Table 2). *Cx. pipiens* shows the largest overall gain in average thermal suitability, with its mean relative R_0_(T) increasing by 0.21 (on a scale from 0 to 1). In contrast, *Cx. quinquefasciatus* and *Cx. tarsalis* exhibit more modest gains of 0.15 and 0.06, respectively. Notably, under the high-emission scenario, both *Cx. quinquefasciatus* and *Cx. tarsalis* show declines in mean R_0_(T) from mid- to late-century, indicating potential thermal constraint at elevated temperatures. By contrast, *Cx. pipiens* continues to gain thermal suitability across both future periods, reaching its peak average R_0_(T) under late-century, high-emission conditions. Spatial projections of environmental risk (Figure 3) for urban and agricultural areas reveal more nuanced, vector species-specific responses, which we expand upon in the Discussion. These responses underscore the importance of integrating both climate and land-use projections when anticipating future WNV risk, as environmental suitability for vectors will not shift uniformly across landscapes.

**4. Discussion**

As climate and land-use change accelerate, understanding the ecological and social factors that shape WNV transmission is increasingly critical for anticipating and mitigating emerging public health threats. Our study demonstrates that temperature and landscape structure, particularly homogenous or transitional zones between urban and agricultural areas, have a consistent but complex relationship with WNV risk. We suggest that these mixed-use environments support overlapping distributions of vectors, birds, and humans, especially in vulnerable communities (4,26,27,50). By investigating current socio-ecological indicators alongside projected vector species-specific, land cover and temperature-dependent transmission model, we present a biologically grounded and complementary framework for understanding disease risk in a changing environment.

Our models show that land-use heterogeneity, suitable temperature for transmission, and human exposure collectively contribute to spatial variation in WNV incidence across US counties. While earlier studies have identified elevated risk in semi-urban or peri-urban zones, our work is among the first to examine national-scale edge effects, supporting prior regional results that WNV incidence is elevated in mixed environments (20,26,27,50). We hypothesize that these mixed zones combine suitable habitat, moderately competent bird hosts, and high enough human population density to drive focal WNV hotspots (24,26). We also find high risk in more homogenous landscapes, likely mirroring the importance of occupational and socioeconomic factors (15–17). Agricultural employment, a proxy for outdoor occupational exposure to *Cx. tarsalis*, showed a positive association with WNV, though further research is needed to understand underreporting and access to care in these populations (16). In urban settings, our findings align with established associations between socioeconomic disadvantage and WNV incidence (15,16,21), potentially mediated by local conditions such as poor drainage, waste accumulation, inadequate housing structure, and limited vector control, all conducive to *Cx. pipiens* proliferation (21,51). While our social vulnerability index detected a risk threshold, its aggregated nature limits mechanistic interpretation (Text S4). Subcounty analyses incorporating specific housing metrics (e.g., infrastructure age, air conditioning access) would likely improve explanatory power, especially in counties with high inequality (15,52). Prior insights from economically diverse Orange County, California underscores the importance of incorporating fine-scale socioeconomic and infrastructure into WNV incidence models, as disparities in infrastructure maintenance, such as the prevalence of neglected swimming pools, can significantly amplify disease risk in lower-income communities (15). Our bird-related findings align with previous research, suggesting that greater richness of competent bird species may be associated with reduced human WNV incidence (22,53). However, our bird metric is limited in that it does not account for seasonal abundance or host-feeding preferences of *Culex* mosquitoes under natural conditions. For example, while both American robins and American crows are competent hosts in laboratory settings, robins are more frequently fed upon by *Culex* mosquitoes in the field (25). We highlight the host-feeding preference of *Culex* species as important areas for future research to aid in our understanding of WNV dynamics (Table 3).

Future WNV risk is unlikely to be spatially uniform, as each *Culex* species, and important bird hosts, respond differently to climate and land-use change due to distinct thermal physiologies and habitat preferences (54). While national averages of temperature-dependent transmission potential suggest broad trends over time (Table 2), these summaries can obscure important regional heterogeneity. Spatial projections of environmental risk (Figure 3) across urban and agricultural areas reveal nuanced, vector species-specific patterns. Much of the nation’s agricultural land is projected to remain thermally suitable; however, *Cx. tarsalis*, which as the broadest thermal tolerance (20.1°C), may experience localized declines in habitat suitability, particularly in regions like the Central Plains where agricultural land is projected to be converted. *Cx. quinquefasciatus*, common in the southern US, has the narrowest thermal breadth (12.7°C) and is expected to see reduced thermal suitability in parts of the south, including current endemic hotspots such as California and Arizona, where future temperatures may exceed its upper thermal limit (31.8°C) (12). *Cx. pipiens*, which has the highest upper thermal limit (34.9°C) and is widely distributed in northern urban areas, is projected to expand its range, particularly in areas undergoing urban development (30,31,55,56). These mosquito species-specific responses emphasize the importance of incorporating both climate and land-use projections when assessing future WNV risk, as environmental risk depends on the interaction between temperature thresholds and habitat availability. While our projections assume fixed thermal limits, evolutionary adaptation or behavioral plasticity in mosquitoes could shift these outcomes over time (57). The resulting county-level risk maps, which integrate temperature-dependent transmission and vector habitat suitability, provide a spatially explicit tool to support targeted surveillance and vector control under future environmental change.

From a public health perspective, our findings highlight further research directions to identify strong mechanistic links, particularly involving social vulnerability and occupational exposure, that can inform local interventions. Our results suggest that improving infrastructure in residential areas and addressing occupational risk could meaningfully reduce environmental WNV exposure, even as climate suitability increases in the US (58). Practical interventions such as improving water management, upgrading housing infrastructure, and conducting outreach to at-risk occupational groups, especially agricultural workers, may help mitigate spillover in ecologically suitable regions (15,52). Targeted management of agricultural landscapes also offer opportunities to reduce risk, for example, by optimizing irrigation practices to minimize standing water and reduce mosquito breeding habitat, particularly in arid regions where irrigation can sustain vector populations well beyond the rainy season (7,8). As urbanization continues to reshape the American landscape, there is a real risk that WNV transmission could be exacerbated without intentional, proactive planning (23,45). Newly urbanized areas, particularly in the Central Great Plains, are of special concern for *Cx. pipiens*-driven transmission given their overlap with existing WNV hotspots (4,10,17). As projected declines in agricultural land cover may reduce habitat suitability for *Cx. tarsalis*, concurrent urbanization and warming temperatures are likely to favor *Cx. pipiens*, indicating a shift in dominant transmission pathways rather than a uniform change in overall environmental suitability. In these regions, urban design that limits mosquito breeding habitat could reduce disease risk (26,51,58). Our results show the value of integrating socio-ecological and climatic data to develop forward-looking, locally-grounded public health strategies in the face of ongoing environmental change.

While our study offers novel insights into the spatial ecology of WNV on a county-level scale across the US, several caveats and limitations must be acknowledged. First, our analyses parse risk of WNND incidence at the county level on average across the period since the virus has invaded the US—a scale too coarse to capture local relationships between land cover, birds, vectors, and humans and to capture weather anomalies that drive temporal variation. This likely explains the dominance of the spatial term and weaker model performance in regions such as the Rocky Mountains and Central Plains, where temporally dynamic processes (e.g., water availability and bird migration) play a key role. Although drought is an important climatic driver of WNV risk, it was not significant in our final analysis. This is likely because we included spatial controls that could encompass local drought variability. Drought could play a more important role in models that explicitly incorporate its temporal variability and use finer (subcounty) spatial resolution to represent vector-host dynamics (2). Notably, our static bird metric does not capture seasonal dynamics of bird migration, which is important for focal WNV hotspots (24,25). Mosquito feeding behavior is another critical consideration (Text S1); however, empirical data are limited and highly localized. In addition, our use of the social vulnerability index, while informative for hypothesis generation, cannot identify mechanisms of exposure. We examined more targeted indicators that directly relate to physical conditions (Text S3) but found the county scale to be too coarse to capture the localized influence of these variables. Finally, our future projections assume a limited number of climate scenarios, and no changes in bird or human exposure metrics, which introduce uncertainty; therefore, our results should be interpretative as relative, not absolute, risk estimates.

Our findings highlight the value of mechanistic, landscape-informed models that integrate species ecology and socio-economic context, as well as unique regional factors, are important to understand WNV incidence. Because the majority of WNV infections are subclinical, scalable socio-ecological frameworks are essential for proactive prediction and intervention. Advances in high-resolution climate and land-use projections, when paired with local metrics of human exposure, can refine vector control strategies under future change. Continued investment in interdisciplinary approaches will be essential to reducing the burden of WNV and other vector-borne diseases.

**Tables**

**Table 1.** GAM results for county-level WNV incidence. The percentage of deviance explained (%) indicates the proportion of the model’s deviance attributed to each covariate, as estimated using the `gam.hp` package. Note that all covariates were initially modeled as splines, but any covariates with edf < 2 (indicating a relationship that is approximately linear) were then fit as linear terms. Linear terms are reported as estimated coefficients with standard errors (SE), t-values, and p-values. Smooth terms are summarized by estimated degrees of freedom (edf), reference degrees of freedom (df), F-statistic, and p-values.

| **Covariate** | **Estimate** | **SE** | **t-value** | **p-value** | **%** |
| --- | --- | --- | --- | --- | --- |
| Intercept | -0.79 | 0.023 | -34.23 | < 2.00 x 10^-16^ |  |
| Prop. employed in ag | 0.12 | 0.019 | 6.68 | 3.17 x 10^-11^ | 3.34 |
| % of population aged ≥65 | 0.22 | 0.020 | 10.86 | < 2.00 x 10^-16^ | 2.97 |
| Total competent bird species | -0.22 | 0.068 | -3.18 | 0.002 | 2.14 |
|  | **edf** | **DF** | **F-statistic** | **p-value** | **%** |
| s(longitude, latitude)^b^ | 27.58 | 19.17 | 109.43 | < 2.00 x 10^-16^ | 75.61 |
| s(average peak-season R_0_(T))^a^ | 7.89 | 8.68 | 20.00 | < 2.00 x 10^-16^ | 7.94 |
| s(urban-agricultural edge density)^a^ | 6.09 | 7.27 | 4.64 | 3.22 x 10^-5^ | 5.58 |
| s(social vulnerability index)^a^ | 2.92 | 3.65 | 6.82 | 5.67 x 10^-5^ | 3.02 |
| Model fit: n = 2,105; adjusted R^2^ = 0.72; deviance explained = 72.3%  ^a^ cubic spline smooth; ^b^Gaussian process smooth | | | | |  |

**Table 2.** Average temperature-driven transmission suitability, R_0_(T) values, across all continental US counties during peak transmission season (April through October) for each *Culex* species under current and future climate scenarios. Projections are for mid-century (2045-2074) and late-century (2075-2100) under two emission scenarios (SSP2-4.5 and SSP5-8.5). Values (mean ± standard deviation) reflect species specific thermal suitability across all counties, based on CMIP6 climate model estimates.

|  |  | **SSP2-4.5**  **(intermediate-emission)** | | **SSP5-8.5**  **(high-emission)** | |
| --- | --- | --- | --- | --- | --- |
| **Species** | *Current* | *Mid* | *Late* | *Mid* | *Late* |
| ***Cx. pipiens*** | 0.36 ± 0.22 | 0.45 ± 0.23 | 0.49 ± 0.22 | 0.50 ± 0.22 | 0.57 ± 0.19 |
| ***Cx. tarsalis*** | 0.48 ± 0.22 | 0.53 ± 0.20 | 0.48 ± 0.18 | 0.54 ± 0.17 | 0.51 ± 0.15 |
| ***Cx. quinquefasciatus*** | 0.40 ± 0.22 | 0.46 ± 0.21 | 0.55 ± 0.18 | 0.48 ± 0.18 | 0.47 ± 0.15 |

**Table 3**. Future research priorities to improve spatial models of WNV risk under climate and land-use change.

1. **Refine the spatial and temporal scale** of the analysis to with more finely resolved epidemiological data, to identify predictors of locally elevated risk.
2. **Refine bird-host metrics** by incorporating *Culex* species’ feeding preferences and temporally resolved bird data (e.g., eBird, Breeding Bird Survey) to better capture avian amplification dynamics within a season and across years.

3. **Develop mechanistic residential exposure metrics** at a finer spatial scale (e.g., data from American Community Survey including housing quality, infrastructure age, swimming pool presence, air conditioning access) to better assess mosquito entry into households (i.e., residential risk), particularly in socially vulnerable communities.

4. **Integrate land cover and population projections** into transmission models to anticipate how urban expansion and demographic change will reshape future WNV exposure patterns.

5. **Model water dispersal patterns** on the landscape, including the effects of irrigation and urban stormwater infrastructure, to better identify where mosquito breeding habitats form and persist.

6. **Establish a standardized, nationwide database of *Culex* abundance** that is readily publicly available using data from counties with active vector control programs, to support consistent and scalable vector surveillance.

7. **Account for evolutionary responses** of *Culex* vectors and WNV to climate change, including potential shifts in thermal tolerance and vector competence under warming conditions.
8. **Incorporate the impacts of variation in WNV exposure and immunity** in humans and bird populations.

**References**

1.	Crowder DW, Dykstra EA, Brauner JM, Duffy A, Reed C, Martin E, et al. West Nile Virus Prevalence across Landscapes Is Mediated by Local Effects of Agriculture on Vector and Host Communities. PLOS ONE. 2013 Jan 30;8(1):e55006. doi:10.1371/journal.pone.0055006

2.	Paull SH, Horton DE, Ashfaq M, Rastogi D, Kramer LD, Diffenbaugh NS, et al. Drought and immunity determine the intensity of West Nile virus epidemics and climate change impacts. Proc R Soc B Biol Sci. 2017 Feb 8;284(1848):20162078. doi:10.1098/rspb.2016.2078 PubMed PMID: 28179512; PubMed Central PMCID: PMC5310598.

3.	Kilpatrick AM. Globalization, Land Use, and the Invasion of West Nile Virus. Science. 2011 Oct 21;334(6054):323–7. doi:10.1126/science.1201010

4.	DeGroote JP, Sugumaran R. National and Regional Associations Between Human West Nile Virus Incidence and Demographic, Landscape, and Land Use Conditions in the Coterminous United States. Vector-Borne Zoonotic Dis. 2012 Aug;12(8):657–65. doi:10.1089/vbz.2011.0786

5.	Moore TC, Tang X, Brown HE. Assessing the Relationship Between Entomological Surveillance Indices and West Nile Virus Transmission, United States: Systematic Review. Vector-Borne Zoonotic Dis. 2025 May;25(5):317–28. doi:10.1089/vbz.2024.0072

6.	Skaff NK, Cheruvelil KS. Fine-scale wetland features mediate vector and climate-dependent macroscale patterns in human West Nile virus incidence. Landsc Ecol. 2016 Sep 1;31(7):1615–28. doi:10.1007/s10980-016-0346-1

7.	Ward MJ, Sorek‐Hamer M, Henke JA, Little E, Patel A, Shaman J, et al. A Spatially Resolved and Environmentally Informed Forecast Model of West Nile Virus in Coachella Valley, California. GeoHealth. 2023 Dec;7(12):e2023GH000855. doi:10.1029/2023GH000855

8.	Kovach TJ, Kilpatrick AM. Irrigation increases and stabilizes mosquito populations and increases West Nile virus incidence. Sci Rep. 2024 Aug 28;14(1):1–9. doi:10.1038/s41598-024-70592-3

9.	Schwartz FW, Ibaraki M, Hort HM. Seasonal Bird Migration Could Explain Regional Synchronicity and Amplification in Human West Nile Virus Case Numbers. GeoHealth. 2025;9(3):e2024GH001194. doi:10.1029/2024GH001194

10.	Hort H, Ibaraki M, Schwartz F. Temporal and Spatial Synchronicity in West Nile Virus Cases Along the Central Flyway, USA. GeoHealth. 2023 May 10;7. doi:10.1029/2022GH000708

11.	Barker CM. Models and Surveillance Systems to Detect and Predict West Nile Virus Outbreaks. J Med Entomol. 2019 Oct 28;56(6):1508–15. doi:10.1093/jme/tjz150

12.	Rochlin I, Faraji A, Healy K, Andreadis TG. West Nile Virus Mosquito Vectors in North America. J Med Entomol. 2019 Oct 28;56(6):1475–90. doi:10.1093/jme/tjz146

13.	Riccetti N, Fasano A, Ferraccioli F, Gomez-Ramirez J, Stilianakis NI. Host selection and forage ratio in West Nile virus–transmitting Culex mosquitoes: Challenges and knowledge gaps. PLoS Negl Trop Dis. 2022 Oct 27;16(10):e0010819. doi:10.1371/journal.pntd.0010819

14.	Bowden SE, Magori K, Drake JM. Regional Differences in the Association Between Land Cover and West Nile Virus Disease Incidence in Humans in the United States. Am J Trop Med Hyg. 2011 Feb 4;84(2):234–8. doi:10.4269/ajtmh.2011.10-0134

15.	Harrigan RJ, Thomassen HA, Buermann W, Cummings RF, Kahn ME, Smith TB. Economic Conditions Predict Prevalence of West Nile Virus. PLoS ONE. 2010 Nov 12;5(11):e15437. doi:10.1371/journal.pone.0015437 PubMed PMID: 21103053; PubMed Central PMCID: PMC2980475.

16.	Vargas Campos CA, García-Pérez S, Figuerola J, Martínez-de la Puente J, Polo I, Rodríguez-de-Fonseca B, et al. Comprehensive analysis of West Nile Virus transmission: Environmental, ecological, and individual factors. An umbrella review. One Health. 2025 Jun 1;20:100984. doi:10.1016/j.onehlt.2025.100984

17.	Chuang TW, Hockett CW, Kightlinger L, Wimberly MC. Landscape-Level Spatial Patterns of West Nile Virus Risk in the Northern Great Plains [Internet]. 2012 Apr 1. doi:10.4269/ajtmh.2012.11-0515

18.	Cardenas VM, Jaime J, Ford PB, Gonzalez FJ, Carrillo I, Gallegos JE, et al. Yard flooding by irrigation canals increased the risk of West Nile disease in El Paso, Texas. Ann Epidemiol. 2011 Dec;21(12):922–9. doi:10.1016/j.annepidem.2011.08.001 PubMed PMID: 21943648; PubMed Central PMCID: PMC3258972.

19.	Reisen W, Lothrop H, Chiles R, Madon M, Cossen C, Woods L, et al. West Nile Virus in California. Emerg Infect Dis. 2004 Aug;10(8):1369–78. doi:10.3201/eid1008.040077

20.	Rochlin I, Turbow D, Gomez F, Ninivaggi DV, Campbell SR. Predictive Mapping of Human Risk for West Nile Virus (WNV) Based on Environmental and Socioeconomic Factors. PLOS ONE. 2011 Aug 10;6(8):e23280. doi:10.1371/journal.pone.0023280

21.	Yitbarek S, Chen K, Celestin M, McCary M. Urban mosquito distributions are modulated by socioeconomic status and environmental traits in the USA. Ecol Appl. 2023;33(5):e2869. doi:10.1002/eap.2869

22.	Allan BF, Langerhans RB, Ryberg WA, Landesman WJ, Griffin NW, Katz RS, et al. Ecological correlates of risk and incidence of West Nile virus in the United States. Oecologia. 2009 Jan;158(4):699–708. doi:10.1007/s00442-008-1169-9

23.	Flores-Ferrer A, Suzán G, Waleckx E, Gourbière S. Assessing the risk of West Nile Virus seasonal outbreaks and its vector control in an urbanizing bird community: An integrative R0-modelling study in the city of Merida, Mexico. Beechler BR, editor. PLoS Negl Trop Dis. 2023 May 30;17(5):e0011340. doi:10.1371/journal.pntd.0011340

24.	Hamer GL, Kitron UD, Goldberg TL, Brawn JD, Loss SR, Ruiz MO, et al. Host Selection by Culex pipiens Mosquitoes and West Nile Virus Amplification. Am J Trop Med Hyg. 2009 Feb;80(2):268–78. doi:10.4269/ajtmh.2009.80.268

25.	Kilpatrick AM, Kramer LD, Jones MJ, Marra PP, Daszak P. West Nile Virus Epidemics in North America Are Driven by Shifts in Mosquito Feeding Behavior. PLOS Biol. 2006 Feb 28;4(4):e82. doi:10.1371/journal.pbio.0040082

26.	Ruiz MO, Walker ED, Foster ES, Haramis LD, Kitron UD. Association of West Nile virus illness and urban landscapes in Chicago and Detroit. Int J Health Geogr. 2007 Mar 12;6(1):10. doi:10.1186/1476-072X-6-10

27.	Reisen WK, Carroll BD, Takahashi R, Fang Y, Garcia S, Martinez VM, et al. Repeated West Nile Virus Epidemic Transmission in Kern County, California, 2004–2007. J Med Entomol. 2009 Jan 1;46(1):139–57. doi:10.1603/033.046.0118

28.	Mordecai EA, Caldwell JM, Grossman MK, Lippi CA, Johnson LR, Neira M, et al. Thermal biology of mosquito‐borne disease. Byers J (Jeb), editor. Ecol Lett. 2019 Oct;22(10):1690–708. doi:10.1111/ele.13335

29.	Shocket MS, Verwillow AB, Numazu MG, Slamani H, Cohen JM, El Moustaid F, et al. Transmission of West Nile and five other temperate mosquito-borne viruses peaks at temperatures between 23°C and 26°C. eLife. 2020 Sep 15;9:e58511. doi:10.7554/eLife.58511

30.	Fay RL, Cruz-Loya M, Keyel AC, Price DC, Zink SD, Mordecai EA, et al. Population-specific thermal responses contribute to regional variability in arbovirus transmission with changing climates. iScience. 2024 Jun;27(6):109934. doi:10.1016/j.isci.2024.109934

31.	Belova A, Mills D, Hall R, Juliana ASt, Crimmins A, Barker C, et al. Impacts of Increasing Temperature on the Future Incidence of West Nile Neuroinvasive Disease in the United States. Am J Clim Change. 2017;06(01):166–216. doi:10.4236/ajcc.2017.61010

32.	Gorris ME, Bartlow AW, Temple SD, Romero-Alvarez D, Shutt DP, Fair JM, et al. Updated distribution maps of predominant Culex mosquitoes across the Americas. Parasit Vectors. 2021 Oct 23;14(1):547. doi:10.1186/s13071-021-05051-3

33.	McDonald E. Surveillance for West Nile Virus Disease — United States, 2009–2018. MMWR Surveill Summ. 2021;70. doi:10.15585/mmwr.ss7001a1

34.	McGarigal K, Tagil S, Cushman SA. Surface metrics: an alternative to patch metrics for the quantification of landscape structure. Landsc Ecol. 2009 Mar;24(3):433–50. doi:10.1007/s10980-009-9327-y

35.	Sohl TL, Sayler KL, Bouchard MA, Reker RR, Friesz AM, Bennett SL, et al. Spatially explicit modeling of 1992–2100 land cover and forest stand age for the conterminous United States. Ecol Appl. 2014 Jul;24(5):1015–36. doi:10.1890/13-1245.1

36.	Tigchelaar M, Battisti DS, Spector JT. Work adaptations insufficient to address growing heat risk for U.S. agricultural workers. Environ Res Lett. 2020 Sep 1;15(9):094035. doi:10.1088/1748-9326/ab86f4

37.	Flanagan BE, Gregory EW, Hallisey EJ, Heitgerd JL, Lewis B. A Social Vulnerability Index for Disaster Management. J Homel Secur Emerg Manag. 2011 Jan 5;8(1). doi:10.2202/1547-7355.1792

38.	Kilpatrick AM, LaDeau SL, Marra PP. Ecology of West Nile Virus Transmission and its Impact on Birds in the Western Hemisphere. The Auk. 2007 Oct 1;124(4):1121–36. doi:10.1093/auk/124.4.1121

39.	Ashfaq M, Rastogi D, Kitson J, Abid MA, Kao SC. Evaluation of CMIP6 GCMs Over the CONUS for Downscaling Studies. J Geophys Res Atmospheres. 2022;127(21):e2022JD036659. doi:10.1029/2022JD036659

40.	Pierce DW, Cayan DR, Feldman DR, Risser MD. Future Increases in North American Extreme Precipitation in CMIP6 Downscaled with LOCA. J Hydrometeorol. 2023 May;24(5):951–75. doi:10.1175/JHM-D-22-0194.1

41.	The CMIP6 landscape. Nat Clim Change. 2019 Oct;9(10):727–727. doi:10.1038/s41558-019-0599-1

42.	Hausfather Z, Peters GP. Emissions – the ‘business as usual’ story is misleading. Nature. 2020 Jan 30;577(7792):618–20. doi:10.1038/d41586-020-00177-3

43.	Mordecai EA, Paaijmans KP, Johnson LR, Balzer C, Ben-Horin T, de Moor E, et al. Optimal temperature for malaria transmission is dramatically lower than previously predicted. Ecol Lett. 2013 Jan;16(1):22–30. doi:10.1111/ele.12015 PubMed PMID: 23050931.

44.	Lai J, Tang J, Li T, Zhang A, Mao L. Evaluating the relative importance of predictors in Generalized Additive Models using the gam.hp R package. Plant Divers. 2024 Jul;46(4):542–6. doi:10.1016/j.pld.2024.06.002

45.	Rochlin I, Faraji A, Ninivaggi DV, Barker CM, Kilpatrick AM. Anthropogenic impacts on mosquito populations in North America over the past century. Nat Commun. 2016 Dec 6;7(1):13604. doi:10.1038/ncomms13604

46.	R Core Team. R: A language and environment for statistical computing [Internet]. Vienna, Austria. Available from: https://www.R-project.org/

47.	Wickham H. ggplot2: Elegant Graphics for Data Analysis. 2nd ed. 2016. Cham: Springer International Publishing : Imprint: Springer; 2016. 1 p. (Use R!). doi:10.1007/978-3-319-24277-4

48.	Wickham H, Averick M, Bryan J, Chang W, McGowan L, François R, et al. Welcome to the Tidyverse. J Open Source Softw. 2019 Nov 21;4(43):1686. doi:10.21105/joss.01686

49.	Sambado S, Vasquez V, Cruz-Loya M, Farner J, Fay R, Bents S, et al. Agriculture-urban interfaces, social vulnerability, and climate change shape West Nile virus risk across the United States [Internet]. Dryad; 2025 [cited 2026 Jan 21]. p. 316300120 bytes. Available from: https://datadryad.org/dataset/doi:10.5061/dryad.dz08kps9j doi:10.5061/DRYAD.DZ08KPS9J

50.	Gibbs SEJ, Wimberly MC, Madden M, Masour J, Yabsley MJ, Stallknecht DE. Factors Affecting the Geographic Distribution of West Nile Virus in Georgia, USA: 2002–2004. Vector-Borne Zoonotic Dis. 2006 Mar;6(1):73–82. doi:10.1089/vbz.2006.6.73

51.	LaDeau S, Leisnham P, Biehler D, Bodner D. Higher Mosquito Production in Low-Income Neighborhoods of Baltimore and Washington, DC: Understanding Ecological Drivers and Mosquito-Borne Disease Risk in Temperate Cities. Int J Environ Res Public Health. 2013 Apr 12;10(4):1505–26. doi:10.3390/ijerph10041505

52.	Hernandez E, Torres R, Joyce AL. Environmental and Sociological Factors Associated with the Incidence of West Nile Virus Cases in the Northern San Joaquin Valley of California, 2011–2015. Vector Borne Zoonotic Dis. 2019 Nov 1;19(11):851–8. doi:10.1089/vbz.2019.2437 PubMed PMID: 31211639; PubMed Central PMCID: PMC6818473.

53.	Swaddle JP, Calos SE. Increased avian diversity is associated with lower incidence of human West Nile infection: observation of the dilution effect. PloS One. 2008 Jun 25;3(6):e2488. doi:10.1371/journal.pone.0002488 PubMed PMID: 18575599; PubMed Central PMCID: PMC2427181.

54.	Ryan SJ. Mapping Thermal Physiology of Vector-Borne Diseases in a Changing Climate: Shifts in Geographic and Demographic Risk of Suitability. Curr Environ Health Rep. 2020 Dec;7(4):415–23. doi:10.1007/s40572-020-00290-5

55.	Keyel AC. Patterns of West Nile Virus in the Northeastern United States Using Negative Binomial and Mechanistic Trait‐Based Models. GeoHealth. 2023 Apr 4;7(4):e2022GH000747. doi:10.1029/2022GH000747 PubMed PMID: 37026081; PubMed Central PMCID: PMC10072317.

56.	Harrigan RJ, Thomassen HA, Buermann W, Smith TB. A continental risk assessment of West Nile virus under climate change. Glob Change Biol. 2014;20(8):2417–25. doi:10.1111/gcb.12534

57.	Couper LI, Farner JE, Caldwell JM, Childs ML, Harris MJ, Kirk DG, et al. How will mosquitoes adapt to climate warming? Perry GH, editor. eLife. 2021 Aug 17;10:e69630. doi:10.7554/eLife.69630

58.	LaDeau SL, Allan BF, Leisnham PT, Levy MZ. The ecological foundations of transmission potential and vector-borne disease in urban landscapes. Funct Ecol. 2015;29(7):889–901. doi:10.1111/1365-2435.12487
