# Supplementary material for: Agriculture–urban interfaces, social vulnerability and climate change shape West Nile virus risk across the United States
Source: Biol Lett. 2026 Oct 7;22(10):20250817. doi: 10.1098/rsbl.2025.0817 (PMC13641919; doi:10.1098/rsbl.2025.0817)
Supplement: Supplementary file 1 [file rsbl.2025.0817.zip › rsbl.2025.0817/rsbl20250817_si_003.docx]

***Biology Letters***

**Agriculture-urban interfaces, social vulnerability, and climate change shape West Nile virus risk across the United States**

Samantha B. Sambado^*^, Váleri N. Vasquez, Mauricio Cruz-Loya, Johannah Farner, Rachel L. Fay, Samantha Bents, Joshua E. Lazaro, Talya Shragai, Isabel O. Delwel, Desire I. Uwera Nalukwago, and Erin A. Mordecai

***Corresponding author**: Samantha Sambado, PhD; [ssambado@stanford.edu](mailto:ssambado@stanford.edu), Stanford University, Sanford, California, United States of America

**DATA AVAILABILITY**

**Supplementary Table 3.** Additional information on data availability. This table summarizes metadata for all variables used in the analysis. “Variable ID” corresponds to the term used in the manuscript figures. “Variable details” provide additional information to locate each indicator and its associated data source. The “Temporal scale” column describes the reference period or time range covered by each dataset. Full source attributions are listed below the table.

| **Source** | **Variable ID** | **Variable details** | **Temporal scale** |
| --- | --- | --- | --- |
| 1 | WNV incidence | West Nile virus human neuroinvasive disease average annual incidence per 100,000 population at the county-level | 1999-2023 |
| 2 | Aged ≥ 65 | Percentage of the population aged 65 and older at the county-level | 2015-2020 |
| 3 | Land cover | 16-class land cover annual classifications at 30 m | 2020 |
| 4 | Bird community | Geographic bird species ranges and predicted habitats (individual polygons) | 2001 |
| 5 | Ag. workers | Proportion of the workforce employed in agriculture at the county-level | 2020-2023 |
| 6 | Social index | Social Vulnerability Index (SVI) which relates to demographic and socioeconomic characteristics at the county-level | 2018, 2020, 2022 |
| 7 | CMIP6 temp. | Temperature projections from the Coupled Model Intercomparison Project 6 (CMIP6), specifically the GFDL-CM4 global climate model, statistically downscaled to ~ 6 km using LOCAv2 (Pierce et al. 2014, 2023) | 2015-2020 (current), 2045-2074 (mid-century), 2075-2100 (late-century) |
| 8 | *Culex* habitat | Predicted habitat suitability of predominant *Culex* mosquitoes across the Americas at 30 m (Gorris et al. 2021) (netCDF files per species) | 1990-2020 |
| 9 | FORE-SCE | Projected land cover classifications from USGS Earth Resources Observation and Science Center using the forecasting scenarios of land-use change (FORE-SCE) model at 250 m (Sohl et al. 2014) | 2020 (current), 2050 (mid-century), 2100 (late-century) |

*Databases*

**^1^ArboNET.** “West Nile virus human neuroinvasive disease average annual incidence per 100,000 population by county of residence, 1999-2024*” *Historic Data*, U.S. Centers for Disease Control and Prevention. <https://www.cdc.gov/west-nile-virus/data-maps/historic-data.html>. Accessed 8 August 2025.

**^2^US Census Bureau.** “65 years and over AGE AND SEX” *American Community Survey 5-year 2020*, US Census Bureau. <https://data.census.gov/table/ACSST5Y2023.S0101?q=S0101&g=010XX00US$0500000>. Accessed 8 August 2025.

**^3^National Land Cover Database (NLCD).** “Annual National Land Cover Database Collection” *National Land Cover Database*, US Geological Survey. <https://www.mrlc.gov/data/land-cover-conus-6>. Accessed 8 August 2025.

**^4^Gap Analysis Program (GAP)**. “Species Data” *Gap Analysis Program*, US Geological Survey. (<https://gapanalysis.usgs.gov/apps/species-data-download/>. Accessed 8 August 2025.

**^5^Bureau of Labor Statistics (BLS).** “Quarterly Census of Employment and Wages (QCEW)” *North American Industry Classification System (NAICS)*, Bureau of Labor Statistics.

<https://data.bls.gov/cew/apps/data_views/data_views.htm#tab=Tables>. Accessed 8 August 2025.

**^6^Agency for Toxic Substances and Disease Registry (ATSDR).** “Social Vulnerability Index” *Place and Health – Geospatial Research Program*, US Centers for Disease Control and Prevention. <https://www.atsdr.cdc.gov/place-health/php/svi/index.html>. Accessed 8 August 2025.

**^7^Coupled Model Intercomparison Project 6 (CMIP6).** “LOCA2/NAmer/GFDL-CM4/0p0625deg/r1i1p1f1” *Historical, ssp245, ssp585*, LOCA2. <https://cirrus.ucsd.edu/~pierce/LOCA2/NAmer/GFDL-CM4/0p0625deg/r1i1p1f1/>. Accessed 8 August 2025.

**^8^Culex habitat.** “culexmaxentmodels” *Los Alamos National Laboratory* (*lanl)*, GitHub. <https://github.com/lanl/culexmaxentmodels>. Accessed 8 August 2025.

**^9^FORE-SCE.** “Conterminous United States Land Cover Projections – 1992 to 2100” *Forecasting Scenarios of Land-Use Change (FORE-SCE) under A2 scenarios*, US Geological Survey. <https://www.usgs.gov/special-topics/land-use-land-cover-modeling/science/land-cover-modeling-methodology-fore-sce-model>. Accessed 8 August 2025.

*Peer-review papers*

Gorris, M. E., A. W. Bartlow, S. D. Temple, D. Romero-Alvarez, D. P. Shutt, J. M. Fair, K. A. Kaufeld, S. Y. Del Valle, and C. A. Manore. 2021. Updated distribution maps of predominant Culex mosquitoes across the Americas. Parasites & Vectors 14:547.

Pierce, D. W., D. R. Cayan, D. R. Feldman, and M. D. Risser. 2023. Future Increases in North American Extreme Precipitation in CMIP6 Downscaled with LOCA. Journal of Hydrometeorology 24:951–975.

Pierce, D. W., D. R. Cayan, and B. L. Thrasher. 2014. Statistical Downscaling Using Localized Constructed Analogs (LOCA)*. Journal of Hydrometeorology 15:2558–2585.

Sohl, T. L., K. L. Sayler, M. A. Bouchard, R. R. Reker, A. M. Friesz, S. L. Bennett, B. M. Sleeter, R. R. Sleeter, T. Wilson, C. Soulard, M. Knuppe, and T. Van Hofwegen. 2014. Spatially explicit modeling of 1992–2100 land cover and forest stand age for the conterminous United States. Ecological Applications 24:1015–1036.
